# Bacterial etiology of ocular and periocular infections, antimicrobial susceptibility profile and associated factors among patients attending eye unit of Shashemene comprehensive specialized hospital, Shashemene, Ethiopia

**DOI:** 10.1186/s12886-020-01398-w

**Published:** 2020-03-30

**Authors:** Ahmed Adem Mohammed, Musa Mohammed Ali, Mengistu Hailemariam Zenebe

**Affiliations:** 1 Shashemene Comprehensive Specialized Hospital, Shashemene, Ethiopia; 2 Hawassa University College of Medicine and Health Sciences department of Medical laboratory, Awassa, Ethiopia

**Keywords:** External eye, Infection, Bacteria, Risk factors, Antimicrobial susceptibility pattern

## Abstract

**Background:**

Eye infection is a public health problem in developing countries including Ethiopia. Bacteria are major causative agents of eye infections that can lead to loss of vision. The objective of this study was to determine bacterial etiology of ocular and periocular infections, antimicrobial susceptibility profile and associated factors among patients who visited the eye unit of Shashamane Comprehensive Specialized Hospital (SCSH).

**Method:**

A hospital-based cross-sectional study was conducted at SCSH from September 1, 2018, to March 30, 2019. Specimens from the ocular and periocular areas were collected from a total of 332 patients who visited the eye unit. Specimens were inoculated on blood agar, chocolate agar, MacConkey agar, and mannitol salt agar. Isolated bacteria were identified by a series of biochemical tests using the standard bacteriological method. Antimicrobial susceptibility test was performed according to the Clinical and Laboratory Standard Institute by disk diffusion method. Factors that could be associated with ocular and periocular infection were collected by using structured questionnaire. Data analysis was done using SPSS version 22.0 software package. A *P* value less than 0.05 was considered statistically significant.

**Result:**

Out of the total 332 study participants with ocular and periocular infections, 198(60%) were culture positive. The proportion of Gram-positive and Gram-negative bacteria were 135(68.2%) and 63(31.8%) respectively. Among Gram-positive bacteria, *Staphylococcus aureus* were predominant. Among Gram-negative bacteria, *Escherichia coli* were predominant. Most *S. aureus* were resistant to penicillin.

**Conclusion:**

Majority of ocular and periocular infections in this study were caused by bacteria; Gram-positive bacteria were responsible for most cases.

## Background

The eye is one of the sense organs in humans which is important throughout life for daily activities. The awareness given to eye health and cleanliness is essential. Dust, high temperature, microorganisms, and other factors can lead to eye diseases which may lead to loss of sight [1].

Although the eye can be infected, it is remarkably resistant to colonization and infection by microbes. There is disparity in the type of bacteria that colonize the eye and other parts of the body. Although the eye is remarkably resistant to colonization and infection by microbes, it is prone to infection because the lens and vitreous are avascular and protein-rich structures; thus, ideal media for the proliferation of many pathogenic bacteria. The external part of the eye is susceptible to bacterial, fungal, viral and parasitic infections [2]. Microorganisms can also invade and damage the internal parts of the eye, which often results in loss of vision [2, 3]. The source of eye infection can be exogenous or endogenous [4]. Clinically external eye infections can be presented as conjunctivitis, keratitis, blepharitis, canaliculitis, dacryocystitis, external hordeolum and cellulitis [5]. The clinical signs and symptoms of inflammation of the eyes along with pus are frequently caused by bacteria. Globally, purulent bacterial conjunctivitis is mainly caused by Gram-positive bacteria. The most common causative agents are *Staphylococcus epidermidis*, *Staphylococcus aureus*, *Streptococcus pneumoniae*, and *Haemophilus influenzae* [3]. The microbial etiology and drug susceptibility, as well as resistance profile may differ with geographic location [3].

The common way of transmission of pathogens is the contact with contaminated fingers, eyelids margins, and adjacent skin, from the nasopharynx via the nasolacrimal duct, from infected eye drops or contact lenses and more rarely from the genitals or via the bloodstream [6]. Bacterial eye infection needs immediate treatment. Treatment of bacterial eye infections involves empirical treatment with topical ophthalmic broad-spectrum antibiotic formulations that become a prevailing practice among ophthalmologists and general practitioners. This along with the irrational use of drugs, availability of antibiotics without prescription, has led to the development of resistance to commonly used antibiotics [7].

There are 1.4 million blind children estimated worldwide, of whom about 320,000 live in Sub-Saharan Africa [8]. In Ethiopia, the prevalence of blindness was reported to be 1.6% and about 87.4% of the cases were due to preventable causes, bacterial infection is one of them [9]. Therefore the aim of this study was to determine the etiology of ocular and periocular infections, antimicrobial susceptibility profile and associated factors.

## Methods

### Study design, period and area

A hospital-based cross-sectional study was conducted among patients suspected of ocular and periocular infections at Shashamane Comprehensive Specialized Hospital (SCSH), eye unit from September 1, 2018, to March 30, 2019. SCSH is located in Shashemene town, Kuyera sub-city. Shashemene is located 250 km to the South of Addis Ababa, the capital city of Ethiopia. The Hospital has 267 beds in the inpatient department, five outpatient departments, and other health service delivery units.

### Study population

All patients seeking treatment for an eye infections at SCSH was considered as source population. Patients with signs and symptoms of ocular and periocular infectious were included in the study. Patients on antibiotics were excluded from the study. In this study convenience sampling technique was used.

Sample size was calculated by using a single proportion formula, n = Z^2^ P (1-P)/d^2^; where n = number of study participants, Z = Reliability coefficient (confidence level) which is 95% = 1.96, P = previous prevalence from Southern part of Ethiopia, 21% [10], the margin of error = 0.05, A contingency of 30% was taken. Based on the calculation the sample size was 332.

### Variables

Dependent variables: Bacterial infection of ocular and periocular and Antimicrobial susceptibility profile.

Independent variables: Sociodemographic and clinical data.

### Operational definitions

#### Conjunctivitis

An eye with redness in colour (bloodshot), oedematous, and have whitish discoloration of discharge which is purulent, sub-conjunctival haemorrhage with lesion.

#### Blepharitis

An eye with gritty (sore eye), with crusting on lashes and appears red, lid-margin inflammation or redness, collarettes around the base of each eyelash, the thickening and cloudiness of the clear oil of the meibomian glands, lash loss, itching or a tickling sensation around or on the eyelids and the presence of Demodex mites.

#### Trauma

An eye presented with pain, producing watery, foreign body sensation and sensitive for the light. And any sign of corneal laceration (distorted pupil), feeling something blow in to the eye and looks like red, any stains with fluorescein.

#### Blepharo-conjunctivitis

An eye presented with burning, irritation or itchy sensation, physically appeared redness and dryness of the eyelids, scaly.

#### Dacryocystitis

An eye presented with purulent reflux with medial canthal massage, fever, cellulitis surrounding the affected lacrimal sac, altered visual acuity and pupillary reaction, diplopia loss of peripheral vision.

### Data collection

#### Sociodemographic and clinical data

Socio-demographic data of each study participants were collected by attending nurses using the structured questionnaire. Ocular and periocular examination (clinical data) was obtained by using a slit lamp bio-microscope to identify any focus of infection or inflammation for all study participants by attending ophthalmologist. The diagnosis was recorded and the specimen was collected by attending ophthalmologist from all study participants presented with Ocular and periocular infections. Quality of the sociodemographic and clinical data was ensured by using a structured, pretested questionnaire.

#### Specimen collection

The specimen was collected from eyelids and conjunctiva using a sterile cotton swab moistened with sterile saline. The swab was rolled over the eyelid margin from medial to the lateral side and back again. Pus from lacrimal sac (dacryocystitis) and blepharitis was collected using dry sterile cotton-tipped swab either by applying pressure over the lacrimal sac to allow the purulent material to reflux through the lacrimal punctum or by irrigating the lacrimal drainage system [11, 12]. Two swabs were collected per individual, labeled and transported immediately to the Microbiology Laboratory of SCSH.

#### Culture and identification

One swab was Gram stained to assess the presence of bacteria, its Gram reaction and presence of polymorphouclear cells. The second swab was inoculated on to 5% sheep blood agar, MacConkey agar, chocolate agar and mannitol salt agar (Oxoid, Ltd) and incubated at 37 °C for 24–48 h. The aerobic atmospheric condition was maintained for the MacConkey agar and mannitol salt agar, while the chocolate agar and 5% sheep blood agar were incubated at 5–10% CO_2_ atmosphere. All plates were initially examined for growth after 24 h and cultures with no growth were re-incubated for an additional 48 h.

After pure colonies were obtained, further identification was conducted using standard microbiological techniques, which include Gram stain, colony morphology, and biochemical tests. Gram-negative bacteria were identified by using several biochemical tests such as; kligler iron agar, citrate utilization test, lysine decarboxylase test, urease test, motility test, indole test, oxidase test, tributyrin, X and V factors. Gram-positive bacteria were identified using hemolytic activity on sheep blood agar, catalase test, coagulase test, bile solubility and optochin disk test [2, 13]. The quality of laboratory data was ensured by checking the expiry date of all reagents and culture media, checking the sterility of culture media before use and by conducting performance tests of culture media by using known strains such as *S. aureus* (ATCC 25923), *E. coli* (ATCC 25922) and *Pseudomonas aeruginosa* (ATCC 27853), *H. influenzae* (ATCC 49247), *Neisseria meningitidis* serogroup-A (ATCC 13077), *S. pneumoniae* (ATCC 49619) and *Neisseria gonorrhea* (ATCC 49226).

#### Antimicrobial susceptibility testing

Antimicrobial susceptibility testing was carried out for each identified bacterium using disc diffusion method based on CLSI 2018 guideline [14]. Nine antibiotic disks such as amoxicillin-clavulanic acid (AMC) 20 μg, ceftriaxone (CRO) 30 μg, ciprofloxacin (CIP) 5 μg, trimethoprim sulphametoxazole (SXT) 25 μg, erythromycin (E) 15 μg, gentamicin (CN) 10 μg, tetracycline (TE) 30 μg, chloramaphenicol (CAF) 30 μg penicillin (P) 10 U and clindamycin (DA) 2 μg were used. (Oxoid Ltd., Basingstoke, and Hampshire, UK). Briefly, 3–5 pure colonies of bacteria were transferred into a test tube containing one ml of sterile normal saline and mixed until the suspension becomes homogenous. The suspension was adjusted to 0.5 McFarland standards. The suspension was uniformly inoculated on to Mueller hinton agar (MHA) for non-fastidious organisms. For fastidious organisms such as *Neisseriae species* MHA enriched with 0.5% sheep blood was used and *Haemophilus* test medium (HTM) was used for *H. influenzae*. The antibiotic disks were placed using disc dispenser on the MHA and incubated at 37 °C for 18–24 h and the zone of inhibition around the disc was measured to the nearest millimeter using a graduated caliper in millimeters. The isolates were classified as susceptible, intermediate and resistant according to CLSI guideline [14]. There are no antibiotic susceptibility breakpoints for topical antibiotic therapy, and it is assumed that comparable or higher antibiotic concentrations are achieved in the ocular tissue during topical treatment.

### Data analysis

Data were entered and cleaned by using SPSS version 22.0 software. All variables were subjected to descriptive and inferential statistics. A P -value, 95% Confidence Interval (CI), and logistic regression were used to interpret the results. If factors showed a *P* value less than 0.25 in bivariate analysis, it was furthers assess by using multivariate analysis and *P*-value less than 0.05 was considered as statistically significant.

## Results

### Sociodemographic data

In the current study, a total of 332 patients seeking treatment for eye infection at SCSH were included; there were no non-respondents. From the total study participants, 177 (53.3%), 133 (40.1%) and 223 (67.2%) were males, in 18–39 years age group and from rural areas respectively. Most of the study participants were students and married (Table 1).

**Table 1:** Sociodemographic characteristics of study participant presented with ocular and periocular infections at Eye Unit of Shashemene Comprehensive Specialized Hospital, September 1, 2018 to March 30, 2019 (*N* = 332)

Variables	Frequency	Percent
**Sex**		
Male	177	53.3%
Female	155	46.7%
**Age Range**		
0–2	17	5.1%
3–11	53	15.9%
12–17	39	11.8%
18–39	133	40.1%
≥ 40	90	27.1%
**Residence**		
Urban	109	32.8%
Rural	223	67.2%
**Occupation**		
Student	126	37.9%
Farmer	69	20.8%
Merchant	12	3.6%
Civil servant	56	16.9%
House Wife	34	10.2%
Not applicable^a^	35	10.5%
**Marital Status**		
Single	61	18.4%
Divorced	1	0.3%
Married	168	50.6%
Not applicable^a^	102	30.7%
**Educational Status**		
Illiterate	27	8.1%
Elementary School	114	34.3%
High School	75	22.6%
College and Above	81	24.4%
Not applicable^a^	35	10.5%

^a^Under age

### Clinical data

Among 332 study participants assessed, the proportions of clinical finding were as follows: conjunctivitis 109 (32.8%), dacryocystitis 76 (22.9%), blepharitis 60 (18.1%), trauma 48 (14.5%), and blephero-conjunctivitis 39 (11.8%). 91 (83.5%) of conjunctivitis, 10 (20.8%) of trauma, 35 (46.1%) of dacryocystitis was caused by bacteria (Table 2).

**Table 2:** Distribution of bacterial isolates across different clinical presentation among study participants presented with ocular and periocular infections at Eye Unit of Shashemene Comprehensive Specialized Hospital, September 1, 2018 to March 30, 2019 (*N* = 332)

Types of clinical presentations	Clinical presentation	Frequency of bacteria among clinical presentation
	n (%)	n (%)
Conjunctivitis	109 (32.8)	91 (83.5)
Dacryocystitis	76 (22.9)	35 (46.1)
Blepharitis	60 (18.1)	36 (60)
Trauma	48 (14.5)	10 (20.8)
Blephero-conjunctivitis	39 (11.8)	26 (66.7)

### Bacterial etiology of ocular and periocular infections

Out of 332 study participants who were examined for ocular and periocular infection, 198(59.6%) were culture positive, mixed infection was not found. Among the total bacteria isolated, 135(68.2%) and 63(31.8%) were Gram positive and Gram negative bacteria respectively. *S. aureus* was the predominant bacteria (Table 3). The predominant bacterium among almost all clinical presentation was *S. aureus* except for dacryocystitis where the predominant bacteria were *S. aureus* and Coagulase Negative *Staphylococci* (CoNS) (Table 4).

**Table 3:** Distribution of bacteria isolated from study participants with ocular and periocular infections who visited Eye Unit of Shashemene Comprehensive Specialized Hospital based on their Gram reaction, September 1, 2018 to March 30, 2019 (*N* = 198)

Bacteria based on Gram reaction	Isolated bacteria	n (%)
**Gram positive bacteria**	*S. aureus*	74 (37.4)
	CoNS	57 (28.8)
	*S. pneumoniae*	4 (2)
**Gram negative bacteria**	*E. coli*	17 (8.6)
	*Klebsiella pneumoniae*	9 (4.6)
	*Moraxella* spp.	8 (4)
	*Citrobacter* spp*.*	7 (3.5)
	*N. gonorrhoeae*	6 (3)
	*H. influenzae*	6 (3)
	*N. meningitidis*	4 (2)
	*Pseudomonas* spp.	3 (1.5)
	*Proteus mirabilis*	3 (1.5)
Total		198

**Table 4:** Types of bacteria isolated across different clinical presentation among patients with ocular and periocular infections at Shashemene Comprehensive Specialized Hospital Eye Unit, September 1, 2018 to March 30, 2019

Bacterial isolates	Types of clinical presentation				
	Conjunctivitis n (%)	Blepharitis n (%)	Blepharo-conjunctivitis n (%)	Dacryocystitis n (%)	Trauma n (%)
*S. aureus*	28 (30.8)	17 (47.2)	17 (65.4)	5 (14.3)	7 (70)
CoNS	19 (20.9)	13 (36.1)	6 (23.1)	17 (48.6)	2 (20%)
*S. pneumoniae*	2 (2.2)	1 (2.8)	1 (3.9)	–	–
*N. gonorrhoeae*	5 (5.5)	1 (2.8)	–	–	–
*N. meningitidis*	3 (3.3)	–	–	1 (2.9)	–
*Moraxella spp.*	5 (5.5)	–	1 (3.9)	2 (5.7)	–
*Pseudomonas* spp*.*	2 (2.2)	–	–	1 (2.9)	–
*H. influenzae*	4 (4.4)	–	–	2 (5.7)	–
*E. coli*	14 (15.4)	1 (2.8)	–	1 (2.9)	1 (10%)
*K. pneumoniae*	4 (4.4)	3 (8.3)	1 (3.9)	1 (2.9)	–
*P. mirabilis*	2 (2.2)	–	–	1 (2.9)	–
*Citrobacter* spp*.*	3 (3.3)	–	–	4 (11.4)	–
Total bacteria	91 (45.9)	36 (18.2)	26 (13.1)	35 (17.7}	10 (5.1%)

Denominator is number of bacteria

### Antimicrobial susceptibility profile

From 135 Gram positive-bacteria 124 (91.9%), 120 (88.9%), and 114 (84.4%), were susceptible to gentamicin, clindamycin, and erythromycin respectively. Among 74 *S. aureus,* 69 (93.2%) and 57 (77%) were resistant to penicillin and tetracycline respectively. Majority of CoNS were resistant to penicillin 56 (98.2%) and tetracycline 50 (87.7%). All *S. pneumoniae* isolates were susceptible to penicillin (Table 5).

**Table 5:** Antimicrobial susceptibility profile of Gram-positive bacteria isolated from study participants with ocular and periocular infections at Shashemene Comprehensive Specialized Hospital Eye Unit, September 1, 2018, to March 30, 2019 (*N* = 135)

Bacteria isolated	Antibiotic Pattern	Antibiotics tested							
		CAF	SXT	CN	TE	E	P	DA	CIP
*S. aureus* *n* = 74	S	53 (71.6%)	51 (68.9%)	72 (97.3%)	2 (2.7%)	61 (82.4%)	4 (5.4%)	66 (89.2%)	63 (85.1%)
	I	18 (24.3%)	16 (21.6%)	1 (1.4%)	15 (20.3%)	5 (6.8%)	1 (1.4%	6 (8.1%)	7 (9.5%)
	R	3 (4.1%)	7 (9.5%)	1 (1.4%	57 (77%)	8 (10.8%)	69 (93.2%)	2 (2.7%)	4 (5.4%)
CoNS *n* = 57	S	44 (77.2%)	40 (70.2%)	49 (85.9%)	3 (5.3%)	49 (85.9%)	–	54 (94.7%)	45 (78.9%)
	I	9 (15.8%)	3 (5.3%)	4 (7%)	4 (7%)	2 (3.5%)	1 (1.8%)	1 (1.8%)	4 (7%)
	R	4 (7%)	14 (24.6%)	4 (7%)	50 (87.8%)	6 (10.5%)	56 (98.3%)	2 (3.5%)	8 (14%)
*S. pneumoniae* *n* = 4	S	3 (75%)	1 (25%)	4 (100%)	3 (75%)	4 (100%)	4 (100%)	ND	ND
	I	–	–	–	–	–	–	ND	ND
	R	1 (25%)	3 (75%)	–	1 (25%)	–	–	ND	ND

*CoNS Coagulase Negative Staphylococci, CAF* Chloramaphenicol, *SXT* Trimethoprim-Sulphametoxazole, *CN* Gentamicin, *TE* Tetracycline, *E* Erythromycin, *P* Penicillin, *DA* Clindamycin, *CIP* Ciprofloxacin, *ND* Not Done. *S* Susceptible, *I* Intermediate, *R* Resistance

Among 63 Gram-negative bacteria isolated 62 (98.4%), and 60(95.2%) were susceptible to ciprofloxacin, and ceftriaxone respectively. All *N. gonorrhoeae* isolates were susceptible to ciprofloxacin, ceftriaxone, tetracycline, and penicillin. All *N. meningitidis* were susceptible to trimethoprim-sulphametoxazole, ciprofloxacin and ceftriaxone. 2/17(11.8%), 2/9(22.2%), 3/3(100%) of *E. coli, K. pneumoniae,* and *P. mirabilis* were resistant to amoxicillin-clavulanic acid (Table 6).

**Table 6:** Antimicrobial susceptibility profile of Gram-negative bacteria isolated from participants with ocular and periocular infections study participants with infections at Shashemene Comprehensive Specialized Hospital Eye Unit, September 1, 2018 to March 30, 2019 (*N* = 63)

Bacteria isolated	Antibiotic Pattern	Antibiotics							
		AMC	SXT	CIP	CRO	CN	CAF	TE	P
*N. gonorrhoeae* *n* = 6	S	ND	ND	6 (100%)	6 (100%)	ND	ND	6 (100%)	6 (100%)
	I	ND	ND	–	–	ND	ND	–	–
	R	ND	ND	–	–	ND	ND	–	–
*N. meningitidis* n = 4	S	ND	4 (100%)	4 (100%)	4 (100%)	ND	2 (50.0%)	ND	ND
	I	ND	–	–	–	ND	2 (50.0%)	ND	ND
	R	ND	–	–	–	ND	–	ND	ND
*Moraxella* spp*.* *n* = 8	S	6 (75.0%)	4 (50.0%)	7 (87.8%)	7 (87.8%)	7 (87.8%)	7 (87.8%)	6 (75.0%)	3 (37.5%)
	I	1 (12.5%)	2 (25.0%)	–	–	–	1 (12.5%)	1 (12.5%)	–
	R	1 (12.5%)	2 (25.0%)	1 (12.5%)	1 (22.2%)	1 (22.2%)	–	1 (12.5%)	5 (62.7%)
*Pseudomonas* spp*.* *n* = 3	S	3 (100%)	–	3 (100%)	3 (100%)	–	3 (100%)	–	ND
	I	–	–	–	–	3 (100%)	–	–	ND
	R	–	3 (100%)	–	–	–	–	3 (100%)	ND
*H. influenzae* *n* = 6	S	3 (50.0%)	4 (66.6%)	6 (100%)	6 (100%)	ND	6 (100%)	4 (66.6%)	ND
	I	3 (50.0%)	1 (16.7%)	–	–	ND	–	–	ND
	R	–	1 (16.7%)	–	–	ND	–	2 (33.3%)	ND
*E. coli* *n* = 17	S	9 (52.9%)	5 (29.4%)	17 (100%)	15 (88.2%)	17 (100%)	14 (82.4%)	1 (5.9%)	ND
	I	6 (35.3%)	1 (5.9%)	–	1 (5.9%)	–	1 (5.9%)	2 (11.8%)	ND
	R	2 (11.8%)	11 (64.7%)	–	1 (5.9%)	–	2 (11.8%)	14 (82.4%)	ND
*K. pneumoniae* *n* = 9	S	6 (66.7%)	6 (66.7%)	9 (100%)	9 (100%)	9 (100%)	8 (88.9%)	2 (22.2%)	ND
	I	1 (11.1%)	–	–	–	–	1 (11.1%)	1 (11.1%)	ND
	R	2 (22.2%)	3 (33.3%)	–	–	–	–	6 (66.7%)	ND
*P. mirabilis* *n* = 3	S	–	3 (100%)	3 (100%)	3 (100%)	3 (100%)	3 (100%)	3 (100%)	ND
	I	–	–	–	–	–	–	–	ND
	R	3 (100%)	–	–	–	–	–	–	ND
*Citrobacter* spp. *n* = 7	S	6 (85.7%)	6 (85.7%)	7 (100%)	7 (100%)	6 (85.7%)	4 (57.1%)	7 (100%)	ND
	I	–	–	–	–	–	2 (28.6%)	–	ND
	R	1 (14.3%)	1 (14.3%)	–	–	1 (14.3%)	1 (14.3%)	–	ND

*AMC* Amoxicillin-clavulanic Acid, *SXT* Trimethoprim-sulphametoxazole, *CIP* Ciprofloxacin, *CRO* Ceftriaxone, *CN* Gentamicin, *CAF* Chloramaphenicol, *TE* Tetracycline, *P* Penicillin, *ND* Not Done. *S* Susceptible, *I* Intermediate, and *R* Resistance

### Factors associated with ocular and periocular infection

None of the factors were significantly associated with ocular and periocular infections (*P* > 0.05). The proportion of bacterial eye infection among study participants with 0–2, 3–11, 12–17, 18–39, ≥ 40 age groups in years were 14(82.3%), 44(83%), 26(66.7%), 73(54.9%), 41(45.6%) respectively. The proportion of bacteria eye infection in rural and urban area were 74(67.9%) and 124(55.6%) respectively. The proportion of bacterial eye infection among participants with repeated infections was 100(50.5%) and among non-repeated infection were 98(49.5%). The proportion of bacterial eye infection among participants with no formal education, elementary, high school, kindergarten, College and above were 12(44.4%), 73(64%), 36(48%), 32(91.4%), 45(55.6%) respectively. The proportion of bacterial eye infection among participants with surgery was 4 (1.2%) without surgery was 194(98%).

## Discussion

The prevalence of culture-positive ocular and periocular infections caused by bacteria found in this study, 59.6%, is in line with studies conducted in various parts of Ethiopia [9, 15, 16]. Our finding is low compared to the report from India (88%) [17], Nigeria (74.9%) [18] and Southern Ethiopia (74.7%) [19]. But it is higher than report from Bangalore (34.5%) [2], Gondar (47.4%) [20] and Addis Ababa (54.2 and 54.9%) [21, 22]. Addis Ababa (54.9%) [22]. The difference can be attributed to geographic location, study period, study population, sanitary condition and laboratory method used. In the current study we did not diagnose eye infection caused by *Chlamydia trachomatis*; this could have caused low prevalence compared to other studies. Overall the prevalence of culture positive ocular and periocular infection in our study is comparable with finding from other parts of Ethiopia [21, 22], but it is low compared to another study from Ethiopia [4]. Gram positive bacteria were predominant in our study like report from [21, 22].

In this study, Gram-positive cocci were the most common isolates (68.2%) which is in line with other studies from Ethiopia [4, 23] and other countries [8, 24, 25]. The finding is low compared to a report from other part of Ethiopia (93.7%) [16]. In the current study, the predominant bacterial isolates were *S. aureus* (37%) followed by CoNS (29%). The finding of this study is comparable with previous studies conducted in Ethiopia [4, 15, 19, 20], Nigeria [8] and India [17].

The proportion of Gram-negative bacteria isolated, (31.8%) in this study is high compared to report from Ethiopia [15, 16, 22]. Among Gram-negative bacteria isolated in the present study, *E. coli* (8.6%) was the most prevalent followed by *K. pneumoniae* (4.6%) and *Moraxella* species 4%). *N. gonorrhea* was also isolated from 6 patients (5 from those with conjunctivitis and 1 from those with blepharitis) suggesting contamination of the eye from the genital area. The high proportion of *E. coli* in this study may indicate fecal contamination of the eye. The finding of the current study is in line with study from Nigeria [8].

Conjunctivitis was the dominant type of clinical presentation (32.8%) observed in this study followed by dacryocystitis (22.9%), blepharitis (18.1%), trauma (14.5%) and Blepharo-conjunctivitis (11.7%). In other studies conjunctivitis was reported to be predominant [16]. The proportion of conjunctivitis found in this study is lower than report from Addis Ababa, Ethiopia (40.5%), the share of bacteria in causing conjunctivitis is comparable to our study (83.5%) [22]. In the current study, *S. aureus* was the most common isolates in all clinical presentation. This finding is similar to a report from India [2].

In this study, the majority of bacteria were resistant to tetracycline and penicillin, while most of them were susceptible to ciprofloxacin. This finding is in agreement with the study conducted in Gondar, Ethiopia [20], Jimma, Ethiopia [23] and Uganda [10]. The reason for increased resistance to penicillin and tetracycline may be prior exposure of the isolates to these antibiotics. Moreover, these antibiotics are common and patients can access them easily with low price and often can be purchased without prescription over the counter in different pharmacies [2].

The majority (77%) of *S. aureus* were resistant to tetracycline and to penicillin (93.2%%); however, 97% were susceptible to gentamicin. A similar finding was reported from other part of Ethiopia [22]. However, low susceptibility (71.9%) to gentamicin [15] and high susceptibility to penicillin was reported from other parts of Ethiopia [16].

Like *S. aureus*, most of CoNS (98.3%) were resistant to penicillin; similarly high resistance to penicillin was reported from Ethiopia [15, 22]. Unlike other studies, majority of CoNS were resistant to tetracycline [15, 22]. The rate of resistance to clindamycin was high compared to finding from other parts of Ethiopia [22]. All *S. pneumoniae* isolated in this study were susceptible to penicillin, erythromycin, gentamicin; this is not in line with other studies [16, 22].

In contrast to another study from Ethiopia [15], all *E. coli* isolates in this study were susceptible to ciprofloxacin and gentamicin. 11.8% of *E. coli* were resistant to amoxicillin-clavulanic acid. All *K. pneumoniae* isolates in this study were susceptible to ciprofloxacin**,** ceftriaxone, and gentamicin. Seven (77.8%) of them were resistant to ampicillin and 22.2% were resistant to amoxicillin-clavulanic acid, this is in partial agreement with Getahun et al. [15] report.

In this study, none of the factors were significantly associated with ocular and periocular infections caused by bacteria (*P* < 0.05]. However, most bacteria were isolated were from participants within the 3–11 age group, those who reside in rural, and those in kindergarten school. Our finding is not comparable with other studies. A report from other parts of Ethiopia indicated a significant association between being farmer and external eye infection caused by bacteria [16]. But other study did not report significant association between factors measured and external eye infection caused by bacteria [22]. According to Getahun et al. [15] previous use of antimicrobials and duration of present illness was significantly associated with bacterial eye infection.

### Limitation of the study

The lack of reagents limited the diagnosis of Chlamydia infections. As we used convenience sampling technique selection bias was not avoided and the study population was not representative of all bacterial eye infection in the study area. Identification of the bacteria in this study does not necessarily mean that the isolated bacteria were responsible for the infection/inflammation.

## Conclusions

In the current study the most prevalent clinical presentation was conjunctivitis followed by Dacryocystitis. From 332 study participants with ocular periocular infections, 59.6% were culture positive. Gram-positive bacteria were the most prevalent with *S. aureus* taking the largest share followed by CoNS. Most Gram positive-bacteria were resistant to Penicillin and Tetracycline. None of the factors were significantly associated with external eye infection caused by bacteria.

## Data Availability

The datasets used and analyzed in this study are available from the corresponding author on reasonable request.

## Abbreviations

SCSH: Comprehensive Specialized Hospital
CLSI: Clinical Laboratory Standard Institute
MHA: Muller Hinton Agar
CoNS: Coagulase Negative Staphylococci
